# The Acute Effect of Resistance Exercise with Blood Flow Restriction with Hemodynamic Variables on Hypertensive Subjects

**DOI:** 10.2478/hukin-2014-0092

**Published:** 2014-11-12

**Authors:** Joamira P. Araújo, Eliney D. Silva, Julio C. G. Silva, Thiago S. P. Souza, Eloíse O. Lima, Ialuska Guerra, Maria S. C. Sousa

**Affiliations:** 1 Associate Program on Graduate Program in Physical Education UPE / UFPB, João Pessoa, Paraíba, Brazil.; 2 Federal Institute of Education Science and Technology of Ceara-Campus Juazeiro do Norte, Brazil.; 3 Kinanthropometry and Human Development Laboratory, Federal University of Paraiba, João Pessoa/PB, Brazil.

**Keywords:** strength training, vascular occlusion, blood pressure, heart rate, hypertension

## Abstract

The purpose of this study was to analyze systolic blood pressure (SBP), diastolic blood pressure (DBP) and the heart rate (HR) before, during and after training at moderate intensity (MI, 50%-1RM) and at low intensity with blood flow restriction (LIBFR). In a randomized controlled trial study, 14 subjects (average age 45±9,9 years) performed one of the exercise protocols during two separate visits to the laboratory. SBP, DBP and HR measurements were collected prior to the start of the set and 15, 30, 45 and 60 minutes after knee extension exercises. Repeated measures of analysis of variance (ANOVA) were used to identify significant variables (2 × 5; group × time). The results demonstrated a significant reduction in SBP in the LIBFR group. These results provide evidence that strength training performed acutely alters hemodynamic variables. However, training with blood flow restriction is more efficient in reducing blood pressure in hypertensive individuals than training with moderate intensity.

## Introduction

Resistance exercise (RE) is an activity mode that is commonly indicated as part of the treatment of diseases such as hypertension, aiding in obtaining increased cardiovascular function. The exercise prescription is very important for safety during the execution. Cardiovascular responses can oscillate depending on the variables of each training session, such as intensity or exercise time (Assunção et al., 2007).

Assunção et al. (2007) stated that RE executed at high intensity should have a substantial static component to provoke an increase in peripheral vascular resistance. The occlusion of the vascular lumen causes metabolite accumulation, triggering muscle chemoreceptors to release catecholamines by stimulating the sympathetic nervous system (Assunção et al., 2007). Consequently, there is an increase in the heart rate (HR) and systolic blood pressure (SBP) during the exercise. Other factors can significantly increase the HR and blood pressure (BP) during the session, such as the muscle mass involved in the exercise execution, the breathing pattern and the number of series executed. For example, larger muscle groups present greater responses in BP.

Therefore, it is necessary to identify different types of training that can result in decreased blood pressure and to distinguish which methods are the most effective in people with hypertension. Previous studies by Lisboa et al. (2007) and Carmo et al. (2007) reported the benefits of strength training for health resulting in decreases in high BP. However, this study focused mostly on acute effects, without comparing different types of training.

RE associated with blood flow restriction, which consists of restricting blood flow to the muscle subjected to exercise, has recently gained prominence. Several studies have demonstrated similar hypertrophic responses compared to high-intensity strength training. Despite this result, there is little information about the behavior of hemodynamic variables during that exercise model in the hypertensive population. To this end, verifying the training effects and analyzing these effects in relation to blood pressure and the heart rate while taking into account the intensity of the exercise required investigation.

Research aiming to control most of the variables involved with moderate intensity resistance exercise and low-intensity resistance exercise associated with blood flow restriction may be biased towards understanding the effectiveness of strength training programs and possible differences in behavior of hemodynamic parameters in hypertensive populations. Thus, the purpose of this study was to examine the acute effects of low-intensity resistance training with blood flow restriction (LIBFR) and moderate-intensity resistance (MI) training on blood pressure and the heart rate before, during and after the exercise.

## Material and Methods

### Subjects

Fourteen women from the city of Juazeiro do Norte-CE with similar fitness levels that were not involved with any type of strength training for the previous six months were recruited. The women had a mean age of 45,71 years. Inclusion was limited to subjects aged 60 and younger who were nonsmokers and had been diagnosed with hypertension type 1 according to the patterns of the WHO/ISH (1999). Subjects diagnosed with another type of disease, such as circulatory, cardiac, and respiratory conditions, were excluded. All participants were informed about the risks associated with the research, and written informed consent was obtained from each participant. The study was approved by the Ethics Committee on Human Research of the Federal University of Paraíba (UFPB), João Pessoa (PB), Brazil (Process number - 0380/11).

### Procedures

Upon arriving at the laboratory, the subject’s anthropometry, body composition and muscle strength were evaluated. After that analysis, the subjects performed one of the two types of training (LIBFR or MI). The individuals were instructed to avoid caffeine, medications and exercises on the test day. Measurements of blood pressure and the heart rate were carried out before the start of the test, immediately after the first, second and third sets, and 15, 30, 45 and 60 minutes after each exercise session.

To carry out the anthropometric assessment, we used an adipometer (Harpenden^®^, TBW, São Paulo, Brazil) for skinfold measurements, an anthropometric tape (Cescorf^®^, Porto Alegre, Brazil) for circumference measurements, a digital scale for measuring weight, and a stadiometer (Wiso^®^, model E210, Santa Catarina, Brazil) fastened to the wall to measure height. To obtain the hemodynamic variables, we used a digital heart rate monitor (Polar^®^, Finlândia) to register the heart rate, and a digital blood pressure monitor (Omron ® HEM-705 CP model, Kyoto, Japão). The external compression (mmHg) for blood flow restriction was performed according to the study of Laurentino et al. (2008) using two special sphygmomanometers (18 cm width × 80 cm length) placed around the upper thigh and a portable vascular Doppler device (model DV2001, Medpej^®^, Ribeirão Preto, SP, Brazil) after the application of coupling gel. A metronome (Tagima^®^, São Paulo, Brazil) was used to control the execution time. The exercise was performed using a knee extension machine. A digital stopwatch was used to control the rest periods between sets.

To assess the maximum power, the “*American Society of Exercise Physiologists*” guidelines were followed (Brown and Weir, 2001). The subjects were familiarized with the exercise and with the maximum repetition test of the knee extension exercise prescribed for the training load. To start the maximum repetition test, a general warm up was performed for five minutes using a cycle ergometer set at 25 Watts (ErgoFit^®^, model 167, Pirmasens, Alemanha). After the warm up, a set of stretching exercises for the lower body and a specific warm up were conducted. The specific warm up was composed of two sets; the first one was performed with 50% of the estimated load for the maximum repetition test in eight repetitions, and the second set was performed with 70% of the estimated load in three repetitions. The rest period between the end of the warm up and the execution of the maximum load test was three to five minutes. The load was gradually increased in 5% intervals based on the last warm up session until the maximum repetition load was reached in at least five attempts. The attempts were performed until the moment that the load increase (kg) made knee extension impossible, and the largest load reached on the previous attempt before concentric failure was taken as the maximum repetition test value.

The determination of arterial occlusion pressure was performed in both legs with 3 minutes of break between each measurement. During the sessions of resistance exercise associated with blood flow restriction, 80% arterial occlusion pressure was used. Analysis of the intra-class correlation coefficient (ICC) to arterial occlusion pressure was performed and values in both groups were 0,97 (p<0,01) for the right leg and 0,96 (p<0,01) for the left leg.

Two types of resistance training were performed: moderate-intensity training (MI) and low-intensity training associated with blood flow restriction (LIBFR). The knee extension exercise was performed by both groups. Each group performed three sessions of fifteen repetitions with some differences between groups. For the LIBRF group, the subjects performed the exercise with a load of 30% of a maximum repetition with a sphygmomanometer applied tightly to each leg around the upper thigh and a rest period of 45 seconds between sets. The sphygmomanometer was inflated during the whole exercise protocol. For the MI group, the load used during the test was 80% of a maximum repetition, without the blood flow restriction and with rest periods of 1 minute between sets. Blood pressure and the heart rate were assessed before and at the end of each session and 15, 30, 45 and 60 minutes after the exercise.

### Statistical Analysis

Statistical analyses were performed using analysis of variance (ANOVA) with repeated measures (2 × 5; group × time). Bonferroni post hoc tests were performed on significant interactions. Independent sample t test analysis was performed to compare the body composition and the maximum repetition load test between the groups. Significance was set at p<0.05. The reproducibility of the blood flow restriction pressure values was analyzed by the intra-class correlation coefficient (ICC) using a two-way random model with absolute concordance. Data are presented as the mean ± standard error unless otherwise noted. Statistical software, SPSS, version 16.0 (SPSS, Chicago, IL, USA) for Windows, was used for all analyses.

## Results

**Figure 1:**
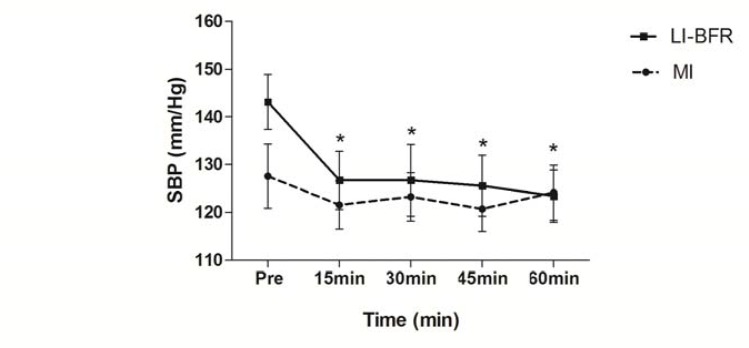
Systolic blood pressure (mm/Hg) measurements pre-exercise and 15, 30, 45 and 60 minutes post-exercise. Values are expressed as the mean ± SE LIBFR: Low-intensity with blood flow restriction; MI: moderated intensity (*) p<0,05 a significant difference for pre, 15, 30, 45 and 60 minutes post-exercise for the LIBFR group.

**Figure 2:**
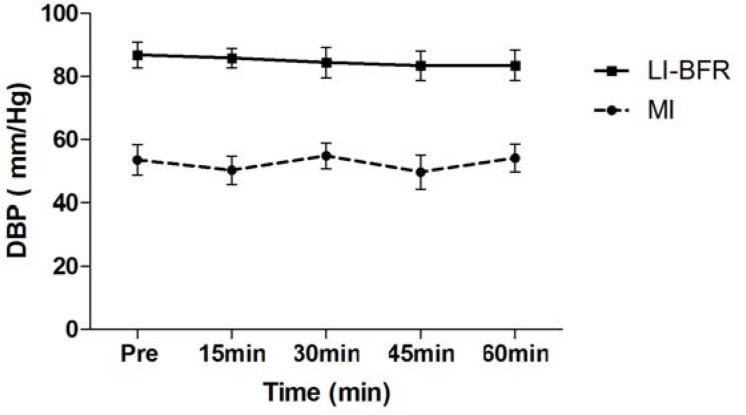
Diastolic blood pressure (mm/Hg) measurements pre-exercise and 15, 30, 45 and 60 minutes post-exercise. Values expressed as the mean ± SE LIBFR: Low-intensity with blood flow restriction; MI: moderated intensity.

**Figure 3:**
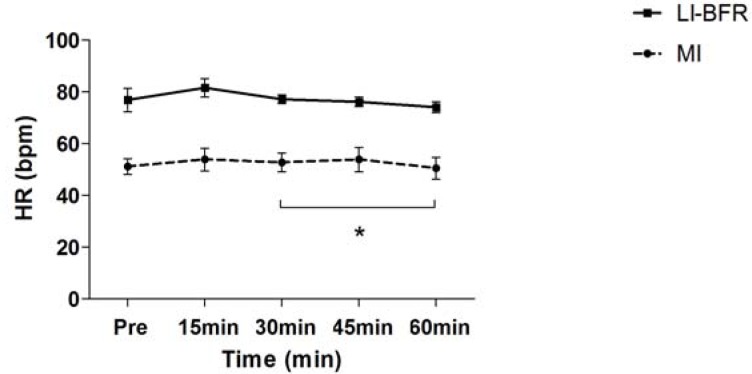
Heart rate (bpm) measured pre-exercise and 15, 30, 45 and 60 minutes post exercise Values expressed as the mean ± SE. LI-BFR: Low-intensity with blood flow restriction; MI: moderated intensity (*) p<0,05 a significant difference for 30 and 60 minutes post-exercise for moderate intensity.

Subject characteristics, including one repetition maximum (1 RM), are presented in Table 1. Table 2 compares hemodynamic responses between sets within each group (MI and LIBFR). Figures 1 and 2 compare SBP and DBP at all time points for both groups. A significant reduction in SBP was observed at all time points (15, 30, 45 and 60 minutes) after training with LIBFR, as shown in Figure 1. There was a significant reduction in the post exercise HR variable between 15 and 60 minutes for the MI group, as shown in figure 3. During exercise, significant differences were found for SBP and DBP in the second set between the groups. A significant increase in the heart rate was observed from the first to the third set in all groups. In the BIFRS group, SBP values were significantly different between the first and third sets compared to the second set. There were significant differences in DBP between the groups only in the second training sets. In the BIFRS group, a significant increase in DBP was observed from the first to the second set. Finally, a significant reduction of DBP between the second and third sets was also observed for the LIBRF group.

## Discussion

To our knowledge, this was the first study to examine hypotensive responses after blood flow restriction exercises in hypertensive subjects. We found that low-intensity strength training with blood flow restriction produced hypotensive responses up to 60 minutes post-exercise. Thus, besides the increase in strength and muscle hypertrophy from the use of LIBFR strength training, it is also possible to achieve decreased SBP responses in hypertensive individuals.

Previous studies have reported that hypotensive effects occurred following resistance exercises (Rossow et al., 2011). Simões et al. (2010)found that the hypotensive effect was greater following strength training exercises with higher loads. Strength training provides benefits in the control of BP, as evidenced in hypertensive and normotensive subjects. Some studies have reported similar results to those described in this report, thereby verifying post-exercise hypotension. Melo et al. (2006) reported a decrease in SBP (−12 +3 mm Hg) and DBP (−6 +2 mmHg) up to 120 minutes after a training session for both the upper and lower body. Costa et al. (2010) also observed hypotension after a session in both trained and untrained elderly hypertensive subjects. According to Polito and Farinatti (2003), it is possible to verify post-exercise hypotension in hypertensive and normotensive individuals. The above data suggest that with regard to an increase in SBP post-exercise, strength training does not present risks when performed acutely. In support of our findings, Pescatello (1991) demonstrated that SBP levels and DBP reduction may persist for approximately 13 hours after acute exercise in hypertensive subjects. This study conjectured that hypotension in hypertensive individuals lasted longer because their baseline values were higher.

Interestingly, BP and HR values during exercise were higher in the BIRFS group compared to the MI group, although hypotensive effects occurred acutely in the BIFRS group. This finding may be explained by the fact that training with BIFRS may be more intense than moderate intensity exercise, so that the subjects’ adaptations seem to mimic exercise sets with high loads.

Corroborating the findings of this study, Suga (2010) suggested that BIFRS protocols with at least 30% of 1 RM may present the same accumulation of intramuscular metabolites as high intensity training. Alternatively, in a study by Rossow et al. (2011) comparing the post exercise hypotensive effect (PEHE) in low intensity resistance training with blood flow restriction and high intensity exercise in active men, it was concluded that PEHE occurred only following high intensity exercise. Perhaps the difference in results from the current study was due to the inclusion of different subject populations and to the use of different exercise intensity protocols.

## Practical Applications

We concluded that the hypotensive effect occurred in the LIBFR group, but not in the MI group, up to 60 minutes post exercise. Thus, strength training with LIBFR may be a more useful method for lowering BP than MI. However, to better understand the hypotensive effect in hypertensive populations using LIBFR strength training, future studies should be conducted with male subjects, and more invasive investigations should be carried out to learn more about the mechanisms that caused these responses.

## Figures and Tables

**Table 1:** Subject characteristics by group (N=14)

	**MI (N=7)**	**LIBFR (N=7)**	**p**
**BMI**	31,2±7,5	30,5±4,4	0,195
**% G**	32,3±5,4	30,0±7,4	0,740
**MM**	40,9±15,1	37,2±5,8	0,054
**HWR**	0,04±0,01	0,02±0,00	0,046*
**1 RM**	46,4±18	58,8±22	0,274

Values are mean ± SD; MI: Moderated intensity; LIBFR: Low-intensity blood flow restriction BMI: Body mass index; % G: Fat-free mass; MM: Muscle mass; HWR: hip-waist ratio; 1 RM: One Repetition maximum.

(*) p<0,05 – a significant difference between the groups (MI and LIBFR)

**Table 2:** Hemodynamic values during exercise

	**1^st^ set**	**2^nd^ set**	**3^rd^ set**
**Hart Rate (bpm)**			
MI	93,5 ± 7,7†	100,5 ± 6,84	108,7 ± 7,3
LIBFR	107,2 ± 7,7†	115 ± 6,8	119,8 + 7,3
**Systolic blood pressure (mm/Hg)**			
MI	141 ± 6	147 ± 10╬	146 ± 10╬
BIFRS	167,4 ± 8*	183 ± 10	173,7 ± 9*
**Diastolic blood pressure (mm/Hg)**			
MI	87 ± 4	86 ± 5╬	87 ± 5
BIFRS	96 ± 5*†	107 ± 4†	88 ± 6

Values are mean ± SE

MI: Moderate intensity; LIBFR: Low-intensity with blood flow restriction

(╬) p<0,005 a significant difference for LIBFR;

(*) p<0,005 a significant difference in 2^nd^ set;

(†) p<0,05 a significant difference in 3^rd^ set
